# Divergent Survival Outcomes With Adjuvant Chemotherapy in Stage IA Ovarian Clear Cell Carcinoma: Insights From the SEER Database

**DOI:** 10.1155/ogi/9983293

**Published:** 2025-10-24

**Authors:** Luping Pan, Yuan Xiang, Jinju Guo, Wei Liu, Xia Wang

**Affiliations:** ^1^Department of Oncology, The Second Affiliated Hospital of Nanchang University, Nanchang, Jiangxi, China; ^2^Nanwai Community Health Service Center, Zhanggong District, Ganzhou, Jiangxi, China; ^3^Department of Oncology, The Shangrao Medical Center, Second Affiliated Hospital of Nanchang University, Shangrao, Jiangxi, China; ^4^The Medical College of Nanchang University, Nanchang, Jiangxi, China; ^5^Jiangxi Key Laboratory of Clinical Translational Cancer Research, Nanchang, Jiangxi, China; ^6^Radiation-Induced Heart Damage Institute of Nanchang University, Nanchang, Jiangxi, China

**Keywords:** adjuvant chemotherapy, ovarian clear cell carcinoma, prognosis, SEER database, stage IA

## Abstract

**Background:**

This study aims to evaluate the impact of adjuvant chemotherapy on cancer-specific survival (CSS) and overall survival (OS) in patients with Stage IA ovarian clear cell carcinoma (OCCC) using data from the Surveillance, Epidemiology, and End Results (SEER) database.

**Methods:**

We conducted a retrospective cohort study utilizing SEER data (2000–2021) to compare the prognosis of Stage IA OCCC patients who received adjuvant chemotherapy versus those who did not. Propensity score matching (PSM) was used to balance baseline characteristics between the groups. Competing risks regression and multivariate Cox regression analyses identified prognostic factors for CSS and OS.

**Results:**

A total of 1422 Stage IA OCCC patients were identified. After PSM, 776 patients (388 in each group) were included. For patients aged ≤ 50 years, chemotherapy was linked to worse CSS (89.5% vs. 96.2%, *p*=0.007) and OS (89.3% vs. 95.9%, *p*=0.008). Conversely, in patients aged > 70 years, chemotherapy was associated with improved CSS (93.0% vs. 81.9%, *p*=0.038) and OS (86.0% vs. 72.4%, *p*=0.006). These trends remained after PSM. Multivariate analysis showed that chemotherapy had little impact on OS and CSS. Subgroup analysis further indicated that chemotherapy negatively affected CSS and OS in patients aged ≤ 50 years.

**Conclusions:**

Adjuvant chemotherapy did not significantly improve survival outcomes in patients with Stage IA OCCC. However, its effects were age-dependent, with older patients (> 70 years) experiencing improved survival, while younger patients (≤ 50 years) exhibited worse outcomes. These findings underscore the importance of individualized treatment strategies for Stage IA OCCC.

## 1. Introduction

Ovarian cancer is the third most common and lethal malignancy of the female reproductive system, characterized by diverse histopathological features [1]. Ovarian clear cell carcinoma (OCCC), a relatively rare histological subtype, accounts for approximately 10% of all ovarian cancer cases. Its incidence varies significantly across ethnic groups, with a higher prevalence observed in East Asia [2–5]. OCCC is typically diagnosed in younger patients and is often detected at an early stage. It is strongly associated with endometriosis, a recognized risk factor for its development. The existing literature indicates that endometriosis is present in 25%–58% of OCCC cases, with most studies reporting its prevalence in over 50% of cases [6–9].

Previous studies have demonstrated that surgical intervention is associated with significantly better outcomes compared to nonsurgical management, regardless of the disease stage [10]. As a result, the initial treatment for OCCC generally involves surgical resection of the tumor and comprehensive staging. Adjuvant chemotherapy may be considered depending on the staging results. However, the role of adjuvant chemotherapy in early-stage OCCC, particularly for Stage IA disease, remains a topic of considerable debate [11–14]. Although the prognosis for Stage IA OCCC is relatively favorable, its biological characteristics and response to chemotherapy differ markedly from other ovarian cancer subtypes, such as serous ovarian carcinoma [15]. Clear cell carcinoma is known for its relative resistance to chemotherapy, which contributes to a higher recurrence rate [16]. This has led to growing attention on the need for adjuvant chemotherapy following surgery.

The impact of adjuvant chemotherapy on the prognosis of patients with Stage IA OCCC is still unclear. While several studies have explored this issue, their findings are inconsistent. OCCC's inherent resistance to chemotherapy, along with its unique biological behavior and prognostic factors, complicates the assessment of chemotherapy's effectiveness [17]. A study by Takano et al. [13] suggests that adjuvant chemotherapy may be unnecessary for patients with Stage I OCCC. This study included 219 patients with Stage I OCCC, 195 of whom received adjuvant chemotherapy (C+ group), while 24 did not (C− group). The C+ group included 77 patients with pT1a tumors and 118 with pT1c, whereas the C− group comprised 18 pT1a and 6 pT1c tumors. No statistically significant differences in progression-free survival or overall survival (OS) were observed between the two groups. In contrast, a study by Nasioudis et al. [18] indicated that adjuvant chemotherapy significantly improved the prognosis of patients with Stage IA and Stage IB ovarian cancer although the improvement for Stage IC patients did not reach statistical significance. This study investigated 2325 patients and found that the 5-year OS of women with Stage IA or IB who received chemotherapy (*n* = 873) was superior to that of those who did not (*n* = 290), with 92.5% and 84% survival rates, respectively (*p* < 0.001). For Stage IC patients, those receiving chemotherapy (*n* = 744) had a favorable trend in OS compared to those not receiving chemotherapy (*n* = 145), with 5-year OS rates of 85.1% and 77.5%, although the difference was not statistically significant (*p*=0.116). The conclusions of these studies are contradictory, and none specifically analyzed patients with Stage IA disease.

Given these conflicting findings, the survival benefits of adjuvant chemotherapy for patients with Stage IA OCCC warrant further investigation to clarify its role in clinical management. This study utilizes the Surveillance, Epidemiology, and End Results (SEER) database to evaluate cancer-specific survival (CSS) and OS in patients with Stage IA OCCC who received adjuvant chemotherapy and conducts multivariate analysis to identify prognostic factors influencing these outcomes.

## 2. Patients and Methods

### 2.1. Study Population and Definitions

Data were extracted from the SEER database using SEER∗STAT software (version 8.4.4), a publicly accessible resource. This study focused on patients diagnosed with Stage IA OCCC between 2000 and 2021. OCCC was defined according to the following ICD-O-3 histology codes: 8310/3 (clear cell adenocarcinoma, NOS), 8313/3 (clear cell adenocarcinofibroma), 8443/3 (clear cell cystadenocarcinoma), and 8444/3 (clear cell cystic tumor, malignant) [14]. Tumor grading followed WHO criteria:

Grade 1: ≤ 5 mitoses/10 HPF with minimal nuclear atypia, Grade 2: 6–10 mitoses/10 HPF with moderate atypia, and Grade 3: > 10 mitoses/10 HPF or severe nuclear pleomorphism. Our search identified 4873 patients with Stage IA OCCC.

The inclusion criteria were as follows: (1) Stage IA disease, (2) age > 18 years, (3) histopathologically confirmed diagnosis of OCCC, (4) available data on treatment modalities (chemotherapy, radiation, and surgery), (5) diagnosis of a single primary malignant neoplasm, and (6) complete follow-up data, including the cause of death. The exclusion criteria included prior adjuvant radiation therapy, lack of surgical intervention, and death within 1 month of diagnosis. After applying these criteria, 1422 patients with Stage IA OCCC were included in the final analysis.

### 2.2. Cohort Definition and Variable Recode

The cohort was divided into two groups based on the receipt of adjuvant chemotherapy following surgery: the chemotherapy group and the nonchemotherapy group. Variables extracted from the SEER database included age at diagnosis, race and ethnicity, histologic subtype, year of diagnosis, laterality, marital status (at diagnosis), lymph node dissection, lymph nodes dissected, tumor size, stage at diagnosis, pathologic grade, surgery types, radiation recode, chemotherapy, survival months, vital status, and cause of death. To assess the impact of age on prognosis, patients were stratified into three age groups using X-tile software (Version 3.6.1), which determined the optimal age cutoffs based on minimizing the log-rank test *p* value.

The resulting age groups were as follows: ≤ 50 years, > 50 and ≤ 70 years, and > 70 years (Figure S1).

### 2.3. Statistical Analysis

The primary endpoints of this study were OS and CSS. OS was defined as the time from diagnosis to death from any cause or to the date of the last follow-up in 2021. CSS was defined as the time from diagnosis to death attributable to ovarian cancer.

Descriptive statistics are presented as medians (range) or counts (percentage). Fisher's exact test was used to compare categorical variables between groups. For continuous variables, the two-sample *t*-test or the Mann–Whitney U test was applied, as appropriate. Propensity score matching (PSM) was performed with a tolerance of 0.001 to balance baseline characteristics between the chemotherapy and nonchemotherapy groups. OS was estimated using Kaplan–Meier survival curves, with comparison between groups assessed via the log-rank test. Multivariate survival analyses were conducted using Cox proportional hazards regression. Competing risks regression (Fine and Gray method) was employed to analyze CSS, accounting for cancer-specific mortality. Statistical significance was defined as a *p* value < 0.05. Data were analyzed using the R programming language (https://www.R-project.org).

## 3. Results

### 3.1. Demographic and Clinical Characteristics

Between 2000 and 2021, a total of 1422 patients with Stage IA OCCC were identified from the SEER database. The demographic and clinical characteristics of these patients are summarized in Table 1. Of these, 967 patients (68.04%) received adjuvant chemotherapy, while 455 (31.96%) did not. The proportion of patients undergoing chemotherapy varied over time, with a notable increase observed from 2010 to 2019 (Figure S2). The cohort was stratified by age into three groups: ≤ 50 years (34.95%), > 50 and ≤ 70 years (46.37%), and > 70 years (18.68%). Significant differences in age distribution were found between the chemotherapy and nonchemotherapy groups (*p* < 0.001). Racially, Black patients constituted the largest subgroup, representing 76.70% in the nonchemotherapy group and 77.35% in the chemotherapy group.

PSM resulted in 776 patients (388 in each group), with no significant differences observed between the chemotherapy and nonchemotherapy groups in terms of age, marital status, race, year of diagnosis, lymph node dissection, number of lymph nodes dissected, or tumor size (*p* > 0.05), ensuring comparability for subsequent survival analyses.

### 3.2. CSS and OS

**Figure 1:**
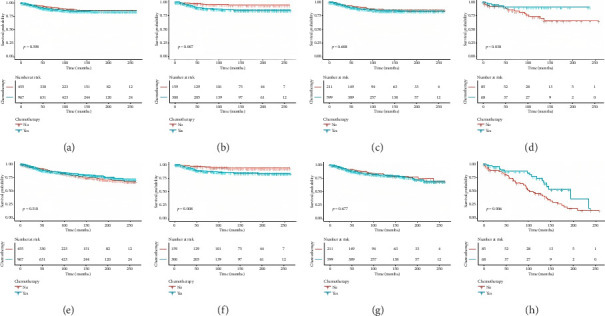
Kaplan–Meier curves for 5-year cancer-specific survival and overall survival in nonchemotherapy and chemotherapy groups across all patients and age subgroups (a–h). (a) All patients: 5-year CSS was 91.6% (nonchemotherapy) vs. 90.3% (chemotherapy) (*p*=0.398). (b) ≤ 50 years: 5-year CSS was 96.2% (nonchemotherapy) vs. 89.5% (chemotherapy) (*p*=0.007). (c) > 50 and ≤ 70 years: 5-year CSS was 91.2% (nonchemotherapy) vs. 90.0% (chemotherapy) (*p*=0.608). (d) > 70 years: 5-year CSS was 81.9% (nonchemotherapy) vs. 93.0% (chemotherapy) (*p*=0.038). (e) All patients: 5-year OS was 87.5% (nonchemotherapy) vs. 89.0% (chemotherapy) (*p*=0.310). (f) ≤ 50 years: 5-year OS was 95.9% (nonchemotherapy) vs. 89.3% (chemotherapy) (*p*=0.008). (g) > 50 and ≤ 70 years: 5-year OS was 88.8% (nonchemotherapy) vs. 87.9% (chemotherapy) (*p*=0.677). (h) > 70 years: 5-year OS was 72.4% (nonchemotherapy) vs. 86.0% (chemotherapy) (*p*=0.006).

The median follow-up was 97 months (range, 1–263) for the nonchemotherapy group and 83 months (range, 1–263) for the chemotherapy group. The 5-year CSS rate was 91.6% (95% confidence interval [CI]: 89.1%–94.1%) in the nonchemotherapy group and 90.3% (95% CI: 88.3%–92.3%) in the chemotherapy group (*p*=0.398; Figure 1(a)). In patients aged ≤ 50 years, the 5-year CSS rate was significantly lower in the chemotherapy group (89.5%, 95% CI: 85.9%–93.3%) compared to the nonchemotherapy group (96.2%, 95% CI: 93.5%–99.1%) (*p*=0.007; Figure 1(b)). Among patients aged > 50 and ≤ 70 years, CSS rates were similar between groups: 90.0% (95% CI: 87.5%–92.7%) in the chemotherapy group and 91.2% (95% CI: 87.5%–95.0%) in the nonchemotherapy group (*p*=0.608; Figure 1(c)). However, for those aged > 70 years, the chemotherapy group exhibited a significant survival advantage, with a 5-year CSS rate of 93.0% (95% CI: 86.9%–99.5%) compared to 81.9% (95% CI: 73.3%–91.3%) in the nonchemotherapy group (*p*=0.038; Figure 1(d)). These findings underscore the age-dependent effects of adjuvant chemotherapy on CSS, suggesting potential harm in younger patients (≤ 50 years) and significant benefit in older patients (> 70 years).

For OS, the 5-year rate was 87.5% (95% CI: 84.7%–90.3%) in the nonchemotherapy group and 89.0% (95% CI: 87.0%–91.0%) in the chemotherapy group, with no significant difference (*p*=0.310; Figure 1(e)). In patients aged ≤ 50 years, the 5-year OS rate was significantly lower in the chemotherapy group (89.3%, 95% CI: 85.7%–93.1%) compared to the nonchemotherapy group (95.9%, 95% CI: 93.1%–98.8%) (*p*=0.008; Figure 1(f)). Among patients aged > 50 and ≤ 70 years, OS rates were similar between groups: 87.9% (95% CI: 85.2%–90.7%) in the chemotherapy group and 88.8% (95% CI: 85.0%–92.8%) in the nonchemotherapy group (*p*=0.677; Figure 1(g)). In contrast, for patients aged > 70 years, the chemotherapy group demonstrated a significant survival advantage, with a 5-year OS rate of 86.0% (95% CI: 79.1%–93.6%) compared to 72.4% (95% CI: 63.4%–82.7%) in the nonchemotherapy group (*p*=0.006; Figure 1(h)). These results suggest a detrimental effect of chemotherapy on OS in younger patients (≤ 50 years) and a clear benefit in older patients (> 70 years).

**Figure 2:**
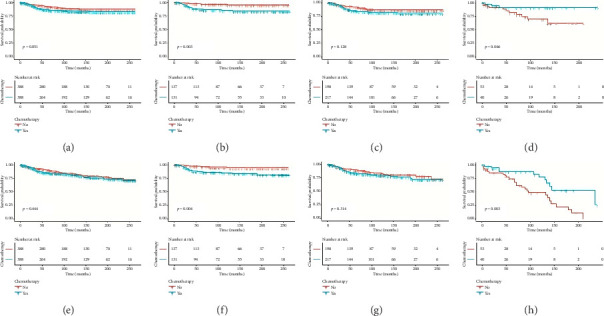
Kaplan–Meier curves for 5-year cancer-specific survival and overall survival in nonchemotherapy and chemotherapy groups among propensity score-matched patients (a–h). (a) All PSM patients: 5-year CSS was 91.7% (nonchemotherapy) vs. 87.7% (chemotherapy) (*p*=0.051). (b) ≤ 50 years: 5-year CSS was 96.7% (nonchemotherapy) vs. 87.4% (chemotherapy) (*p*=0.003). (c) > 50 and ≤ 70 years: 5-year CSS was 90.8% (nonchemotherapy) vs. 86.1% (chemotherapy) (*p*=0.128). (d) > 70 years: 5-year CSS was 77.8% (nonchemotherapy) vs. 92.9% (chemotherapy) (*p*=0.046). (e) All PSM patients: 5-year OS was 88.8% (nonchemotherapy) vs. 87.3% (chemotherapy) (*p*=0.444). (f) ≤ 50 years: 5-year OS was 96.4% (nonchemotherapy) vs. 87.7% (chemotherapy) (*p*=0.004). (g) > 50 and ≤ 70 years: 5-year OS was 88.2% (nonchemotherapy) vs. 85.2% (chemotherapy) (*p*=0.314). (h) > 70 years: 5-year OS was 68.9% (nonchemotherapy) vs. 88.1% (chemotherapy) (*p*=0.003).

After PSM, the results were largely consistent with those before PSM. The 5-year CSS rate in the nonchemotherapy group was 91.7%, and in the chemotherapy group, it was 87.7% (*p*=0.051; Figure 2). Notably, the chemotherapy group showed worse CSS in younger patients (≤ 50 years, 87.4% vs. 96.7%; *p*=0.003), but a significant advantage in those > 70 years (92.9% vs. 77.8%; *p*=0.046). Similarly, for OS after PSM, no significant difference was found overall (*p*=0.444), but chemotherapy again negatively affected younger patients (≤ 50 years) and benefited older patients (> 70 years).

### 3.3. Prognostic Factors for CSS and OS

In a multivariate analysis of the entire cohort (*N* = 1422), chemotherapy did not significantly affect either OS (hazard ratio [HR] = 0.98, 95% CI: 0.74–1.30, *p*=0.902) or CSS (HR = 1.47, 95% CI: 0.96–2.24, *p*=0.075) (Table 2). Age at diagnosis was a significant predictor of OS, with patients aged > 50 to ≤ 70 years (HR = 2.09, 95% CI: 1.46–2.98, *p* < 0.001) and > 70 years (HR = 5.91, 95% CI: 3.86–9.05, *p* < 0.001) showing worse OS compared to those aged ≤ 50 years. Race also significantly influenced both OS and CSS, with White patients demonstrating worse OS (HR = 2.41, 95% CI: 1.36–4.28, *p*=0.002) and a borderline worse CSS (HR = 2.21, 95% CI: 1.00–4.87, *p*=0.050) compared to Black patients. Marital status was another important factor, with married patients showing improved OS (HR = 0.72, 95% CI: 0.52–0.99, *p*=0.046) and CSS (HR = 0.64, 95% CI: 0.43–0.97, *p*=0.034) relative to single or unmarried patients. Lymph node dissection involving more than 10 nodes was associated with better OS (HR = 0.72, 95% CI: 0.52–0.99, *p*=0.045) and CSS (HR = 0.49, 95% CI: 0.32–0.76, *p*=0.002). Tumor laterality also influenced CSS, with right-sided tumors (HR = 1.48, 95% CI: 1.01–2.17, *p*=0.045) and tumors of unknown or other laterality (HR = 4.12, 95% CI: 1.37–12.42, *p*=0.012) associated with worse CSS compared to left-sided tumors. Additionally, higher tumor grade (G3) was linked to worse CSS (HR = 1.69, 95% CI: 1.00–2.83, *p*=0.049). These findings underscore the limited prognostic role of chemotherapy, while highlighting the pivotal influence of age, marital status, lymph node dissection, tumor laterality, and tumor grade on both OS and CSS.

In the PSM cohort (*N* = 330), consistent with the findings from the entire cohort, chemotherapy did not significantly influence OS (HR, 1.09; 95% CI: 0.76–1.55; *p*=0.644) or CSS (HR, 1.71; 95% CI: 0.92–3.18; *p*=0.089) (Table 3). However, the analysis reaffirmed the pivotal roles of non–treatment-related factors in determining survival outcomes. Age at diagnosis remained a strong prognostic factor, with patients aged > 50–70 years showing worse OS (HR, 2.37; *p* < 0.001) and CSS (HR, 2.97; *p*=0.005) compared to those ≤ 50 years, while those aged > 70 years exhibited even poorer outcomes (OS: HR, 7.54; *p* < 0.001; CSS: HR, 2.97; *p*=0.039). Marital status similarly demonstrated prognostic significance, with married patients experiencing better OS (HR, 0.62; *p*=0.028) and CSS (HR, 0.29; *p* < 0.001) compared to single or unmarried individuals. In contrast, divorced or separated patients had worse CSS (HR, 0.12; *p*=0.015), as did widowed patients (HR, 0.24; *p*=0.031). Lymph node dissection involving > 10 nodes continued to be associated with improved CSS (HR, 0.39; *p*=0.019), corroborating findings from the entire cohort. Tumor laterality also influenced outcomes, with patients with other or unknown laterality experiencing significantly worse CSS (HR, 7.33; *p*=0.002). Finally, elevated or undocumented CA-125 levels emerged as strong indicators of poor CSS (elevated: HR, 23.25; *p*=0.009; undocumented: HR, 20.19; *p*=0.010). Taken together, these findings from the PSM cohort not only reinforce the limited prognostic impact of chemotherapy but also highlight the consistent influence of demographic, clinical, and tumor-specific factors such as age, marital status, lymph node dissection, tumor laterality, and CA-125 levels on survival outcomes.

### 3.4. Subgroup Analysis

To investigate the prognostic significance of adjuvant chemotherapy in patients with OCCC, multivariate subgroup analyses were performed to assess CSS and OS across diverse clinicopathological factors.

**Figure 3:**
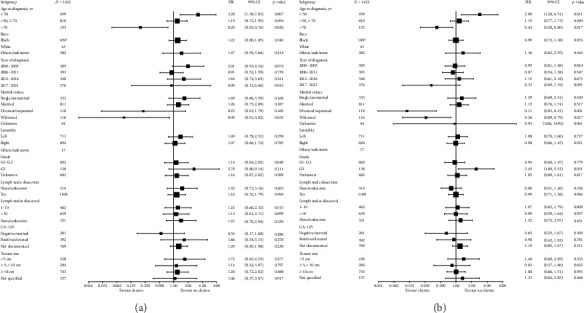
Forest plots of subgroup analyses evaluating the impact of adjuvant chemotherapy on survival outcomes within diverse patient and tumor subgroups. (a) Subgroup analysis of cancer-specific survival. (b) Subgroup analysis of overall survival.

Subgroup analyses for CSS examined the impact of adjuvant chemotherapy across various patient and tumor characteristics (Figure 3(a)). Chemotherapy was associated with significantly worse CSS in patients aged ≤ 50 years (HR, 3.28; 95% CI, 1.38–7.83; *p*=0.007). In contrast, patients aged > 70 years derived substantial survival benefits from chemotherapy, with improved CSS (HR, 0.20; 95% CI, 0.05–0.76; *p*=0.020). Widowed patients demonstrated a significant CSS benefit from chemotherapy (HR = 0.09, 95% CI: 0.01–0.82, *p*=0.032). While not statistically significant, a trend toward worse CSS was noted in patients with high-grade tumors (G3) treated with chemotherapy (HR, 2.70; 95% CI, 0.80–9.14; *p*=0.111). Additionally, chemotherapy did not significantly influence CSS in subgroups defined by lymph node dissection, the number of lymph nodes dissected, or tumor size.

Subgroup analyses for OS evaluated the role of adjuvant chemotherapy across comparable characteristics (Figure 3(b)). Among patients aged ≤ 50 years, chemotherapy was associated with significantly worse OS (HR, 2.89; 95% CI, 1.28–6.51; *p*=0.011). Conversely, patients aged > 70 years experienced notable improvements in OS with chemotherapy (HR, 0.42; 95% CI, 0.20–0.86; *p*=0.017). Divorced or separated patients demonstrated substantial OS benefits (HR, 0.11; 95% CI, 0.03–0.41; *p*=0.001), as did widowed patients (HR, 0.26; 95% CI, 0.09–0.79; *p*=0.017). No significant OS differences were observed in other marital status groups. Conversely, patients with high-grade tumors (G3) demonstrated worse OS outcomes with chemotherapy (HR = 2.45, 95% CI: 1.09–5.52, *p*=0.031). No significant OS differences were observed in other subgroups.

**Figure 4:**
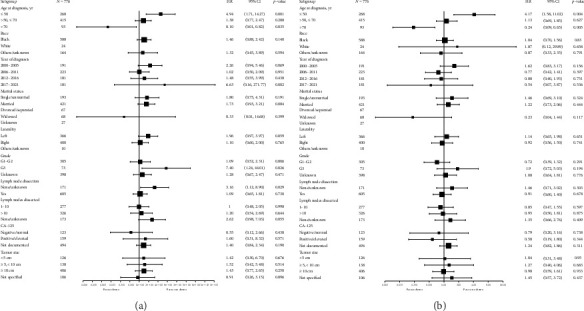
Forest plots of subgroup analyses evaluating the impact of adjuvant chemotherapy on survival outcomes after propensity score matching within diverse patient and tumor subgroups. (a) Subgroup analysis of cancer-specific survival. (b) Subgroup analysis of overall survival.

In the subgroup analysis following PSM, the impact of chemotherapy on CSS demonstrated substantial heterogeneity across various subgroups (Figure 4(a)). Chemotherapy was associated with significantly worse CSS in patients aged ≤ 50 years (HR: 4.94; 95% CI: 1.71–14.27; *p*=0.001), whereas it conferred a significant survival advantage to patients aged > 70 years (HR: 0.10; 95% CI: 0.01–0.82; *p*=0.035), underscoring age as a pivotal determinant of chemotherapy efficacy. Further analysis of other subgroups, including race, year of diagnosis, marital status, tumor laterality, and tumor size, revealed no statistically significant improvements in CSS with chemotherapy (all *p* values > 0.05). However, a notable exception was observed in patients with G3 tumors, for whom chemotherapy was associated with significantly shorter survival (HR: 7.40; 95% CI: 1.24–44.01; *p*=0.026), and those with no or unknown lymph node dissection, who exhibited significantly improved CSS with chemotherapy (HR: 3.16; 95% CI: 1.12–8.90; *p*=0.029).

The subgroup analysis also revealed significant variability in the effect of chemotherapy on OS (Figure 4(b)). Consistent with the findings for CSS, chemotherapy was associated with significantly worse survival in patients aged ≤ 50 years (HR: 4.17; 95% CI: 1.58–11.02; *p*=0.004). In contrast, patients aged > 70 years demonstrated substantial survival benefit with chemotherapy (HR: 0.24; 95% CI: 0.09–0.65; *p*=0.005), reinforcing the critical role of age as a modifier of chemotherapy efficacy.

Among other subgroups, including race, year of diagnosis, marital status, tumor grade, lymph node status, and CA-125 levels, chemotherapy did not result in statistically significant differences in OS (all *p* values > 0.05). Notably, no significant survival differences were observed within the tumor size subgroup across stratified groups.

## 4. Discussion

This study utilized data from the SEER database spanning from 2000 to 2021 to analyze the clinical characteristics, survival outcomes, and associated prognostic factors in patients with Stage IA OCCC who received adjuvant chemotherapy. Of the 1422 identified patients, 68.04% received adjuvant chemotherapy. Our analysis revealed a gradual increase in chemotherapy usage prior to 2019, followed by a noticeable decline in recent years. This trend aligns with the findings of a retrospective study analyzing chemotherapy usage among Stage I ovarian cancer patients in the National Cancer Database (NCDB) from 2004 to 2015, where chemotherapy rates increased from 73.2% (2004–2006) to 84.9% (2013–2015) (*p* < 0.001) [18]. This underscores the ongoing debate in clinical practice regarding the necessity of chemotherapy for early-stage patients.

Contrary to our expectations, we found no significant differences in the 5-year OS and CSS rates between patients who received chemotherapy and those who did not. However, an important age-dependent pattern emerged: younger patients (≤ 50 years) who received chemotherapy had worse survival outcomes, while older patients (≥ 70 years) showed improved survival with chemotherapy. These findings remained consistent even after adjusting for baseline characteristics using PSM. This observation highlights the critical role of age as a determining factor in chemotherapy efficacy.

Our results are consistent with previous studies, suggesting that older ovarian cancer patients often receive less chemotherapy due to concerns over treatment-related toxicity and side effects [19]. However, chemotherapy has been shown to improve survival in this cohort. In contrast, our study found that younger patients (≤ 50 years) did not benefit from chemotherapy and, in fact, showed worse outcomes. This raises the possibility that age-related biological factors, such as tumor biology, immune responses, or the presence of cancer stem cells (CSCs), might influence chemotherapy efficacy in this population [20–22].

Interestingly, the relationship between chemotherapy and survival in younger patients diverges from studies in other cancers, such as breast cancer [20]. For example, breast cancer patients, particularly those with tumors that have higher tumor-infiltrating lymphocyte densities, typically respond better to chemotherapy [20]. Our findings raise the hypothesis that some younger OCCC patients may respond less favorably to chemotherapy; the potential contribution of the tumor microenvironment is speculative and warrants validation in OCCC-specific studies. Additionally, research in osteosarcoma has demonstrated that younger patients often possess genetic mutations associated with chemotherapy sensitivity, while older patients tend to exhibit alterations that contribute to chemotherapy resistance [21].

Moreover, studies on locally advanced rectal cancer (LARC) further support our hypothesis that age-related biological factors, such as immune aging and genetic mutations, may influence chemotherapy outcomes [22]. In younger LARC patients, a higher burden of CSCs has been identified, which may contribute to chemotherapy resistance. This finding mirrors our results, suggesting that age-specific molecular characteristics, including immune response and CSC presence, play a significant role in chemotherapy resistance. Taken together, our findings, along with the existing literature, underscore the importance of considering age as a key determinant of chemotherapy efficacy in OCCC and other cancers.

In addition to age, our study identified several other factors that significantly impacted survival outcomes. These included marital status, lymph node dissection, tumor laterality, tumor grade, and CA-125 levels. Despite chemotherapy not demonstrating a significant effect on survival for Stage IA OCCC patients, these factors should be considered when determining the most appropriate treatment strategies for patients with this rare and aggressive form of ovarian cancer. A holistic approach, accounting for both clinical and molecular factors, is crucial for optimizing patient management and improving prognostic evaluations.

While the body of research on the role of adjuvant chemotherapy in Stage IA OCCC remains limited, several studies have reported conflicting conclusions. For instance, Takada et al. [23] argued that adjuvant chemotherapy is unnecessary for Stage IA OCCC, but their study involved only 73 patients, with only 41.1% receiving chemotherapy. This small sample size limits the generalizability and reliability of their conclusions. Conversely, Oseledchyk et al. [14] conducted a large cohort study using the SEER database, including 1995 patients with Stage I OCCC, and similarly found no significant impact of adjuvant chemotherapy on OS. However, their study did not perform multivariate analysis or PSM, which could lead to confounding biases and impact the validity of their findings.

Other studies have reached differing conclusions. For example, Dinkelspiel et al. [24] categorized 1394 Stage I ovarian cancer patients and found that chemotherapy improved OS in high-risk groups but did not significantly impact CSS. Similarly, Nasioudis et al. [18] reported that adjuvant chemotherapy improved OS for patients with Stage IA and Stage IB ovarian cancer but did not show a statistically significant benefit for Stage IC patients. In contrast, the study by Hogen et al. [12] advocates for the use of adjuvant chemotherapy in all Stage I ovarian cancer patients. However, the methodological limitations in many of these studies, including small sample sizes and lack of multivariate analysis, highlight the need for further research to clarify the role of chemotherapy in Stage IA OCCC.

Notably, the studies by Yin et al. [25] and Bogani et al. [26], both rigorous meta-analyses with large sample sizes, concluded that adjuvant chemotherapy has no impact on the prognosis of Stage IA and Stage IB OCCC patients. However, these studies included overlapping data from the NCDB and SEER databases, which could potentially introduce biases due to duplicated patient records. Given the shared use of data between these databases, caution must be exercised when interpreting their conclusions.

Currently, high-quality evidence for Stage IA OCCC remains limited. Major guidelines (e.g., NCCN and ESMO/ESGO) acknowledge this uncertainty—observation after comprehensive staging may be appropriate for Stage IA, and adjuvant platinum-based chemotherapy is variably recommended across early stages [27]. Our findings add real-world evidence to this debate and, after PSM, support a cautious, individualized approach rather than routine chemotherapy for all Stage IA cases.

Our study specifically focused on Stage IA OCCC, excluding the less common Stage IB patients. This approach helps avoid ambiguities in previous studies on early-stage OCCC, where distinguishing between Stage IC due to ovarian surface involvement and intraoperative rupture has been challenging. Some studies have suggested that patients diagnosed with Stage IC OCCC due to rupture have OS rates similar to those with Stage IA disease [23], whereas those with surface involvement have significantly poorer outcomes. This distinction is critical for accurately assessing prognosis and treatment outcomes in OCCC.

Despite its strengths, our study has limitations. We did not examine the number of chemotherapy cycles, the specific chemotherapy agents used, or their dosages, which may have influenced the outcomes. Additionally, as a retrospective analysis, our data may be subject to selection and information biases. While treatment indication bias cannot be eliminated, the demographic breadth and longitudinal nature of SEER provide unique insights unobtainable from conventional studies. For rare ovarian malignancies, such data remain indispensable until prospective molecularly stratified cohorts emerge. Future prospective studies are needed to further validate and expand on these findings.

In conclusion, our study is the first to highlight the differential impact of adjuvant chemotherapy on survival in Stage IA OCCC across different age groups, revealing that chemotherapy may not benefit younger patients (≤ 50 years) and may even have detrimental effects, while older patients (> 70 years) may experience improved survival outcomes. We also identified several factors, such as marital status, lymph node dissection, tumor laterality, tumor grade, and CA-125 levels, that significantly influence survival. These findings underscore the need for individualized treatment strategies, considering not only age but also tumor characteristics and other patient-specific factors. Further prospective studies are warranted to validate these results and guide clinical decision-making in Stage IA OCCC.

## Figures and Tables

**Table 1:** Patient demographics and associations with adjuvant chemotherapy before and after PSM.

Characteristic	Before propensity score-matched (*N* = 1422)			After propensity score-matched (*N* = 776)		
	No chemo (*N* = 455)	Chemo (*N* = 967)	*p* value	No chemo (*N* = 388)	Chemo (*N* = 388)	*p* value
Age at diagnosis (yr)			< 0.001			0.244
≤ 50	159 (34.95%)	300 (31.02%)		137 (35.31%)	131 (33.76%)	
> 50, ≤ 70	211 (46.37%)	599 (61.94%)		198 (51.03%)	217 (55.93%)	
> 70	85 (18.68%)	68 (7.03%)		53 (13.66%)	40 (10.31%)	
Race			0.204			0.246
Black	349 (76.70%)	748 (77.35%)		290 (74.74%)	298 (76.80%)	
White	9 (1.98%)	34 (3.52%)		9 (2.32%)	15 (3.87%)	
Others/unknown	97 (21.32%)	185 (19.13%)		89 (22.94%)	75 (19.33%)	
Year of diagnosis			< 0.001			0.711
2000–2005	127 (27.91%)	178 (18.41%)		100 (25.77%)	91 (23.45%)	
2006–2011	129 (28.35%)	264 (27.30%)		106 (27.32%)	117 (30.15%)	
2012–2016	102 (22.42%)	246 (25.44%)		94 (24.23%)	87 (22.42%)	
2017–2021	97 (21.32%)	279 (28.85%)		88 (22.68%)	93 (23.97%)	
Marital status			0.003			0.972
Single/unmarried	110 (24.18%)	223 (23.06%)		98 (25.26%)	95 (24.48%)	
Married	239 (52.53%)	572 (59.15%)		211 (54.38%)	210 (54.12%)	
Divorced/separated	35 (7.69%)	83 (8.58%)		31 (7.99%)	36 (9.28%)	
Widowed	55 (12.09%)	61 (6.31%)		35 (9.02%)	33 (8.51%)	
Unknown	16 (3.52%)	28 (2.90%)		13 (3.35%)	14 (3.61%)	
Laterality			0.579			0.526
Left	221 (48.57%)	490 (50.67%)		189 (48.71%)	177 (45.62%)	
Right	227 (49.89%)	467 (48.29%)		193 (49.74%)	207 (53.35%)	
Other/unknown	7 (1.54%)	10 (1.03%)		6 (1.55%)	4 (1.03%)	
Grade			0.291			0.898
G1–G2	181 (39.78%)	421 (43.54%)		155 (39.95%)	150 (38.66%)	
G3	42 (9.23%)	96 (9.93%)		35 (9.02%)	38 (9.79%)	
Unknown	232 (50.99%)	450 (46.54%)		198 (51.03%)	200 (51.55%)	
Lymph node dissection			< 0.001			0.436
None/unknown	132 (29.01%)	182 (18.82%)		81 (20.88%)	90 (23.20%)	
Yes	323 (70.99%)	785 (81.18%)		307 (79.12%)	298 (76.80%)	
Lymph nodes dissected			0.001			0.215
1–10	144 (31.65%)	318 (32.89%)		132 (34.02%)	145 (37.37%)	
> 10	182 (40.00%)	457 (47.26%)		175 (45.10%)	151 (38.92%)	
None/unknown	129 (28.35%)	192 (19.86%)		81 (20.88%)	92 (23.71%)	
CA-125			< 0.001			0.823
Negative/normal	61 (13.41%)	200 (20.68%)		59 (15.21%)	64 (16.49%)	
Positive/elevated	82 (18.02%)	310 (32.06%)		78 (20.10%)	81 (20.88%)	
Not documented	312 (68.57%)	457 (47.26%)		251 (64.69%)	243 (62.63%)	
Tumor size			0.013			0.666
< 5 cm	79 (17.36%)	159 (16.44%)		69 (17.78%)	57 (14.69%)	
≥ 5, < 10 cm	80 (17.58%)	204 (21.10%)		69 (17.78%)	69 (17.78%)	
≥ 10 cm	229 (50.33%)	514 (53.15%)		200 (51.55%)	206 (53.09%)	
Not specified	67 (14.73%)	90 (9.31%)		50 (12.89%)	56 (14.43%)	
Follow-up, median (range), mo	97 (1–263)	83 (1–263)	0.006	96 (1–263)	98.50 (1–263)	0.711

**Table 2:** Multivariate analyses on variables for the prediction of CSS and OS of selected study cohort.

Characteristic (*N* = 1422)	*N* (%)	Overall survival		Cancer-specific survival	
		Hazard ratio (95% CI)	*p* value	Hazard ratio (95% CI)	*p* value
Age at diagnosis (yr)					
≤ 50	459 (32.28%)	1 (reference)	—	1 (reference)	—
> 50, ≤ 70	810 (56.96%)	2.09 (1.46, 2.98)	< 0.001	1.49 (0.93–2.38)	0.099
> 70	153 (10.76%)	5.91 (3.86, 9.05)	< 0.001	1.87 (0.96–3.65)	0.068
Race					
Black	1097 (77.14%)	1 (reference)	—	1 (reference)	—
White	43 (3.02%)	2.41 (1.36, 4.28)	0.002	2.21 (1.00–4.87)	0.050
Others/unknown	282 (19.83%)	1.00 (0.70, 1.42)	0.986	0.95 (0.57–1.57)	0.834
Year of diagnosis					
2000–2005	305 (21.45%)	1 (reference)	—	1 (reference)	—
2006–2011	393 (27.64%)	1.31 (0.93, 1.84)	0.121	1.74 (1.07–2.84)	0.026
2012–2016	348 (24.47%)	1.32 (0.83, 2.09)	0.236	1.60 (0.84–3.05)	0.156
2017–2021	376 (26.44%)	0.74 (0.37, 1.45)	0.380	0.33 (0.12–0.89)	0.029
Marital status					
Single/unmarried	333 (23.42%)	1 (reference)	—	1 (reference)	—
Married	811 (57.03%)	0.72 (0.52, 0.99)	0.046	0.64 (0.43–0.97)	0.034
Divorced/separated	118 (8.30%)	0.56 (0.33, 0.97)	0.038	0.31 (0.12–0.85)	0.022
Widowed	116 (8.16%)	1.01 (0.64, 1.59)	0.973	0.44 (0.19–1.00)	0.050
Unknown	44 (3.09%)	0.93 (0.44, 1.97)	0.848	0.84 (0.32–2.21)	0.724
Laterality					
Left	711 (50.00%)	1 (reference)	—	1 (reference)	—
Right	694 (48.80%)	1.22 (0.94, 1.58)	0.131	1.48 (1.01–2.17)	0.045
Other/unknown	17 (1.20%)	1.94 (0.68, 5.56)	0.217	4.12 (1.37–12.42)	0.012
Grade					
G1–G2	602 (42.33%)	1 (reference)	—	1 (reference)	—
G3	138 (9.70%)	1.03 (0.68, 1.56)	0.904	1.69 (1.00–2.83)	0.049
Unknown	682 (47.96%)	1.13 (0.85, 1.50)	0.403	1.01 (0.66–1.55)	0.951
Lymph node dissection					
None/unknown	314 (22.08%)	1 (reference)	—	1 (reference)	—
Yes	1108 (77.92%)	0.82 (0.44, 1.52)	0.528	1.08 (0.43–2.70)	0.873
Lymph nodes dissected					
1–10	462 (32.49%)	1 (reference)	—	1 (reference)	—
> 10	639 (44.94%)	0.72 (0.52, 0.99)	0.045	0.49 (0.32–0.76)	0.002
None/unknown	321 (22.57%)	1.10 (0.59, 2.05)	0.752	0.92 (0.37–2.31)	0.861
CA-125					
Negative/normal	261 (18.35%)	1 (reference)	—	1 (reference)	—
Positive/elevated	392 (27.57%)	1.14 (0.70, 1.84)	0.598	1.05 (0.51–2.20)	0.887
Not documented	769 (54.08%)	1.14 (0.72, 1.83)	0.573	1.37 (0.67–2.80)	0.396
Tumor size					
< 5 cm	238 (16.74%)	1 (reference)	—	1 (reference)	—
≥ 5, < 10 cm	284 (19.97%)	0.79 (0.51, 1.22)	0.282	1.11 (0.58–2.13)	0.758
≥ 10 cm	743 (52.25%)	0.86 (0.60, 1.25)	0.432	1.15 (0.65–2.03)	0.636
Not specified	157 (11.04%)	1.05 (0.67, 1.65)	0.822	1.49 (0.77–2.87)	0.237
Chemotherapy					
None/unknown	455 (32.00%)	1 (reference)	—	1 (reference)	—
Yes	967 (68.00%)	0.98 (0.74, 1.30)	0.902	1.47 (0.96–2.24)	0.075

**Table 3:** Multivariate analyses on variables for the prediction of CSS and OS of PSM cohort.

Characteristic (*N* = 330)	*N* (%)	Overall survival		Cancer-specific survival	
		Hazard ratio (95% CI)	*p* value	Hazard ratio (95% CI)	*p* value
Age at diagnosis (yr)					
≤ 50	268 (34.54%)	1 (reference)	—	1 (reference)	—
> 50, ≤ 70	415 (53.48%)	2.37 (1.47, 3.83)	0.000	2.97 (1.39, 6.35)	0.005
> 70	93 (11.98%)	7.54 (4.30, 13.23)	< 0.001	2.97 (1.06, 8.36)	0.039
Race					
Black	588 (75.77%)	1 (reference)	—	1 (reference)	—
White	24 (3.09%)	1.06 (0.41, 2.73)	0.910	0.93 (0.18, 4.89)	0.929
Others/unknown	164 (21.13%)	0.86 (0.53, 1.41)	0.555	0.70 (0.32, 1.51)	0.363
Year of diagnosis					
2000–2005	191 (24.61%)	1 (reference)	—	1 (reference)	—
2006–2011	223 (28.74%)	1.30 (0.83, 2.04)	0.257	1.59 (0.80, 3.17)	0.189
2012–2016	181 (23.32%)	1.30 (0.72, 2.36)	0.390	0.86 (0.33, 2.25)	0.756
2017–2021	181 (23.32%)	0.87 (0.36, 2.11)	0.766	0.11 (0.02, 0.60)	0.011
Marital status					
Single/unmarried	193 (24.87%)	1 (reference)	—	1 (reference)	—
Married	421 (54.25%)	0.62 (0.41, 0.95)	0.028	0.29 (0.15, 0.56)	0.000
Divorced/separated	67 (8.63%)	0.59 (0.29, 1.17)	0.131	0.12 (0.02, 0.65)	0.015
Widowed	68 (8.76%)	0.71 (0.39, 1.30)	0.268	0.24 (0.07, 0.88)	0.031
Unknown	27 (3.48%)	0.70 (0.24, 2.01)	0.508	0.38 (0.05, 3.08)	0.368
Laterality					
Left	366 (47.16%)	1 (reference)	—	1 (reference)	—
Right	400 (51.55%)	1.19 (0.84, 1.70)	0.326	1.53 (0.84, 2.78)	0.165
Other/unknown	10 (1.29%)	4.46 (1.29, 15.44)	0.018	7.33 (2.08, 25.82)	0.002
Grade					
G1–G2	305 (39.30%)	1 (reference)	—	1 (reference)	—
G3	73 (9.41%)	1.27 (0.73, 2.21)	0.401	4.45 (2.02, 9.79)	0.000
Unknown	398 (51.29%)	1.11 (0.76, 1.64)	0.587	1.36 (0.63, 2.94)	0.439
Lymph node dissection					
None/unknown	171 (22.04%)	1 (reference)	—	1 (reference)	—
Yes	605 (77.96%)	0.70 (0.25, 1.99)	0.503	2.22 (0.47, 10.46)	0.314
Lymph nodes dissected					
1–10	277 (35.70%)	1 (reference)	—	1 (reference)	—
> 10	326 (42.01%)	0.64 (0.42, 0.98)	0.040	0.39 (0.18, 0.85)	0.019
None/unknown	173 (22.29%)	0.93 (0.32, 2.65)	0.885	1.96 (0.44, 8.71)	0.375
CA-125					
Negative/normal	123 (15.85%)	1 (reference)	—	1 (reference)	—
Positive/elevated	159 (20.49%)	1.06 (0.52, 2.14)	0.880	23.25 (2.19, 247.20)	0.009
Not documented	494 (63.66%)	1.06 (0.56, 2.02)	0.858	20.19 (2.04, 200.21)	0.010
Tumor size					
< 5 cm	126 (16.24%)	1 (reference)	—	1 (reference)	—
≥ 5, < 10 cm	138 (17.78%)	0.96 (0.49, 1.87)	0.893	1.23 (0.28, 5.37)	0.787
≥ 10 cm	406 (52.32%)	1.32 (0.76, 2.30)	0.317	2.03 (0.60, 6.86)	0.255
Not specified	106 (13.66%)	1.48 (0.78, 2.79)	0.227	2.63 (0.70, 9.82)	0.151
Chemotherapy					
None/unknown	388 (50.00%)	1 (reference)	—	1 (reference)	—
Yes	388 (50.00%)	1.09 (0.76, 1.55)	0.644	1.71 (0.92, 3.18)	0.089

## Data Availability

The data files utilized in this study were directly obtained from the SEER website (https://seer.cancer.gov/).

## Nomenclature

OCCC: Ovarian clear cell carcinoma
SEER: Surveillance, Epidemiology, and End Results
CSS: Cancer-specific survival
OS: Overall survival
PSM: Propensity score matching
HR: Hazard ratio
CI: Confidence interval
NCDB: National Cancer Database
LARC: Locally advanced rectal cancer
CSC: Cancer stem cell

## Supporting Information

Supporting Information 1 Figure S1. Age-based grouping of patients using X-tile software. The optimal age cutoff points, determined by X-tile software, divided patients into three groups: ≤ 50 years, > 50 and ≤ 70 years, and > 70 years.

Supporting Information 2 Figure S2. Proportion of patients receiving chemotherapy by year of diagnosis (2000–2021). This graph demonstrates the trend in chemotherapy utilization over time, showing a substantial increase from 2000 to 2019, followed by a modest decline in 2021.
