# Supplementary figures and images for: Divergent Survival Outcomes With Adjuvant Chemotherapy in Stage IA Ovarian Clear Cell Carcinoma: Insights From the SEER Database
Source: Obstet Gynecol Int. 2025 Oct 24;2025:9983293. doi: 10.1155/ogi/9983293 (PMC12578555; doi:10.1155/ogi/9983293)

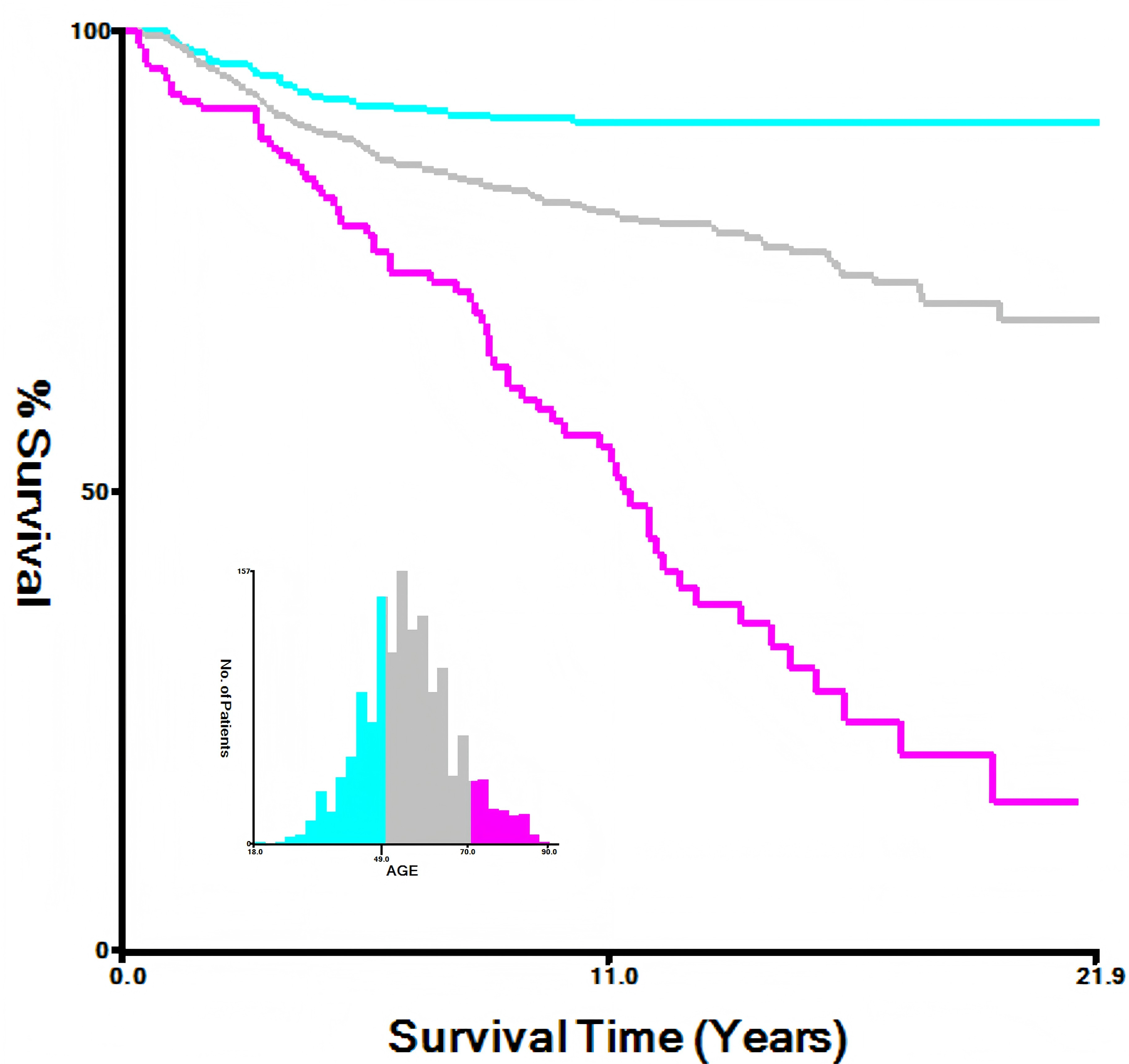

Supplement: Supporting Information 1 — Figure S1. Age-based grouping of patients using X-tile software. The optimal age cutoff points, determined by X-tile software, divided patients into three groups: ≤ 50 years, > 50 and ≤ 70 years, and > 70 years. [file 9983293.f1.pdf]

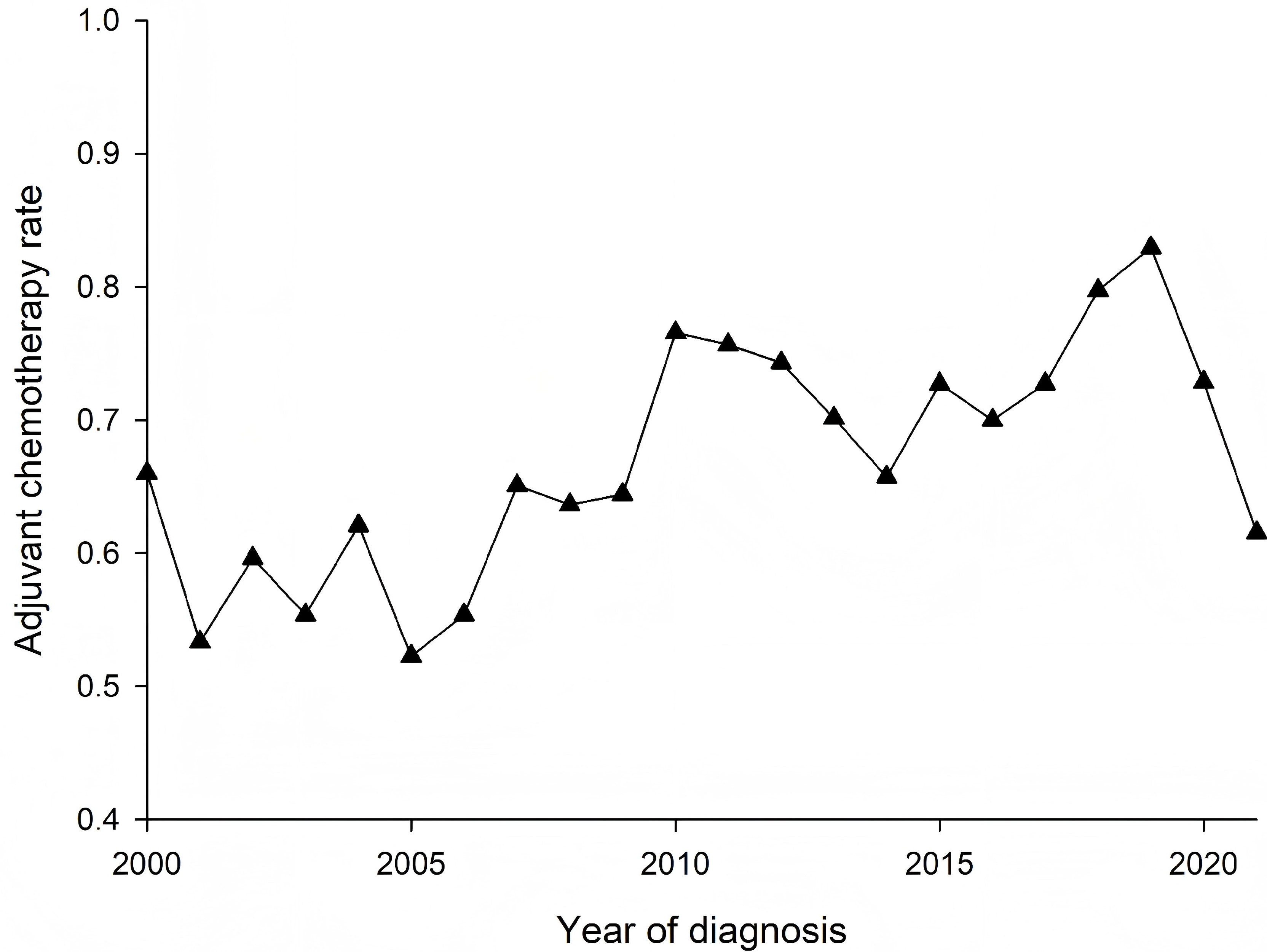

Supplement: Supporting Information 2 — Figure S2. Proportion of patients receiving chemotherapy by year of diagnosis (2000–2021). This graph demonstrates the trend in chemotherapy utilization over time, showing a substantial increase from 2000 to 2019, followed by a modest decline in 2021. [file 9983293.f2.pdf]
